# ID 89 - Análise de Custos das Ocorrências de Incêndios Residenciais e/ou em Edificação (Classe A) no Rio de Janeiro

**DOI:** 10.5327/2237-9622.2025.v34s1.89

**Published:** 2025-11-25

**Authors:** Cristiano Bertolossi Marta, Márglory Fraga de Carvalho, Roberto Carlos Lyra da Silva, Adriana Ferreira Barbosa, Carmem Costa Borges, Marco Antônio Basques

**Keywords:** custo e análise de custo, incêndios, despacho de emergência médica

## Abstract

**Introdução:**

A probabilidade de incêndios no Rio de Janeiro representa 69,9%, com uma incidência de 1,01% e aqueles que envolvem sólidos e inflamáveis (Classe A) são os mais frequentes (58,10%; n=1.381) destacando assim um alto valor agregado à função de bombeiro militar. Promover a conscientização de uma gestão sustentável enquanto empresa pública revela a necessidade de melhor compreensão e adequação de processos de modo a tornar o sistema ainda mais eficiente. Dessa forma, tem-se como problema de pesquisa: Quais contribuições podem ser observadas, a partir de uma análise econômica, aplicada às ocorrências de incêndios (classe A) residenciais e/ou em edificação no Rio de Janeiro? Como objetivos tem-se: Identificar o fluxo de atividades de todo processo de atendimento à incêndio; levantar os custos por etapas e citar as oportunidades gerenciais. A justificativa do estudo está em municiar o gestor na tomada de decisão permitindo reinvestimento nos recursos humanos e materiais, de forma mais assertiva.

**Método:**

Trata-se de uma análise econômica, em saúde do tipo parcial, utilizando-se de uma técnica de micro custeio retrospectiva, num horizonte temporal de um ano, avaliando os componentes de custos do atendimento a incêndio residencial e/ou em edificações (classe A), no ano de 2022, na perspectiva pública. O atendimento foi mapeado e dividido em três etapas, em que o início do processo é marcado pela entrada de ligações na Central 193 (etapa 1), passando pela recepção da ocorrência no quartel e acionamento do comboio para o local (etapa 2) e, por fim, o combate e extinção do foco de incêndio e atendimento às vítimas na cena (etapa 3). Para cada etapa do atendimento foram elencados os itens de custo (diretos e indiretos) e o tempo médio das atividades foram mensurados.

**Resultados:**

O atendimento de incêndio residencial e/ou em edificações, envolveu as atividades desempenhadas por 25 militares, num tempo médio de 01:07:43, apresentou um custo total de R$177.253,17 e um custo/hora de R$1.230,92. A etapa 3 apresentou o maior custo/hora (R$741,25), o maior tempo (00:37:17), envolveu o uso de recursos médicos e não médicos em cena, representando 60,22% do custo total do atendimento, o equivalente a R$106.740,66 . Já a etapa 2 representou apenas 17,71% do custo total (R$31.399,59). O menor percentual de custo direto se deu na etapa 2, com o consumo de combustível pelas viaturas representando menos de 1% do custo total (0,67%).

**Conclusão:**

Apesar da etapa 2 apresentar o maior número de subitens de custo, seu custo total é inferior quando comparado ao custo total da etapa 1 (R$ 31.399,59 versus R$ 39.112,92) e isso pode estar associado ao número de militares em cada etapa, suas especialidades, seus quadros e seus tempos de serviço na Corporação. Extrapolando, os custos com trotes em 2022 representou 3,85% (R$37.223.165,70) do custo total com incêndios classe A (R$ 965.852.523,33) no mesmo período. Os custos com trotes seriam suficientes para adquirir entre 74 e 124 ambulâncias totalmente equipadas anualmente, aumentando assim a capacidade de resposta e atendimento à população. Conclui-se que qualquer instituição que busque resultados de eficiência, eficácia e efetividade precisa conhecer o custo real de seus processos para então compará-los com as demais alternativas disponíveis e as análises econômicas são essenciais neste contexto.

